# Potential prophylactic efficacy of mast cell stabilizers against COVID-19 vaccine-induced anaphylaxis

**DOI:** 10.1186/s12948-021-00162-9

**Published:** 2021-12-13

**Authors:** Itsuro Kazama

**Affiliations:** School of Nursing, Miyagi University, 1-1 Gakuen, Taiwa-cho, Kurokawa-gun, Miyagi, 981-3298 Japan

**Keywords:** Coronavirus disease 2019 (COVID-19), Vaccines, Anaphylaxis, Mast cell stabilizer

## Abstract

To fight against coronavirus disease 2019 (COVID-19), the vaccination is currently the most effective approach. However, in addition to common systemic side effects, the vaccines can cause serious allergic reactions or anaphylaxis. In anaphylaxis, the exposure to the allergen causes a sudden release of chemical mediators from mast cells, for which adrenaline is the drug of first choice. In our previous basic studies, in addition to adrenaline, anti-allergic drugs (olopatadine, loratadine, tranilast and ketotifen), antibiotics (clarithromycin), corticosteroids (hydrocortisone and dexamethasone) and certain food constituents (caffeine and catechin) inhibited the process of exocytosis and showed their effectiveness as highly potent mast cell stabilizers. In these studies, since mast cells were pre-incubated with these drugs or the food constituents before exocytosis was induced, the findings strongly indicated their prophylactic efficacy in stabilizing mast cells. Considering such pharmacological properties of these commonly prescribed medications or the food constituents, their prophylactic use may potentially be beneficial in preventing anaphylaxis caused by COVID-19 vaccination.

## To the editor,

The Italian Society of Allergology, Asthma and Clinical Immunology (SIAAIC) has recently produced a series of indications to manage patients with allergic disorders during the Coronavirus disease 2019 (COVID-19) pandemic [1]. However, regarding the COVID-19 vaccine-induced allergic reactions, there are not enough clinical or experimental data to draw the guidelines to manage or prevent them.

COVID-19 causes severe acute respiratory syndrome and continues to spread around the world. Some patients develop fatal pneumonia or multiple organ failure due to generalized thrombotic microangiopathy as the result of cytokine storm [2]. Others, regardless the severity of the disease, suffer from post-COVID syndrome which is characterized by persistent respiratory or systemic symptoms after recovery [3]. Recently, due to the predominancy of new severe acute respiratory syndrome coronavirus 2 (SARS-CoV-2) variants that are highly contagious, the number of seriously ill patients with COVID-19 and their mortality rate are increasing [4, 5].

To fight against the virus and help stop the pandemic, the vaccination is currently the most effective approach [6]. According to recent studies, the administration of COVID-19 mRNA vaccines actually protected the individuals from the infection [7], prevented the onset of symptoms and reduced the severity of the disease with more than 90% efficacy rates [8, 9]. However, in addition to common systemic side effects that are usually self-limited, including fever, headache, generalized fatigue and arthralgia [10], the vaccines, though rare, can cause serious allergic reactions or anaphylaxis, which is an acute, potentially life-threatening multisystem syndrome [11–14]. In such cases, polyethylene glycol (PEG), one the ingredients of the vaccine, is considered to be responsible, since some patients were actually proven to be allergic to PEG [15, 16].

Concerning the mechanisms, the exposure to allergens, such as medications, foods and insect stings, causes a sudden release of chemical mediators from mast cells, including histamine, serotonin and leukotrienes. In the acute treatment, adrenaline, a non-specific adrenergic receptor agonist, is the drug of first choice, since the stimulation of α- and β_1_-adrenergeic receptors reverses the cardiovascular collapse and ameliorates the airway obstruction [17]. Additionally, adrenaline quickly attenuates the serious allergic reaction fundamentally, since the stimulation of β_2_-adrenergeic receptors directly suppresses further release of chemical mediators from mast cells [18, 19].

**Fig. 1:**
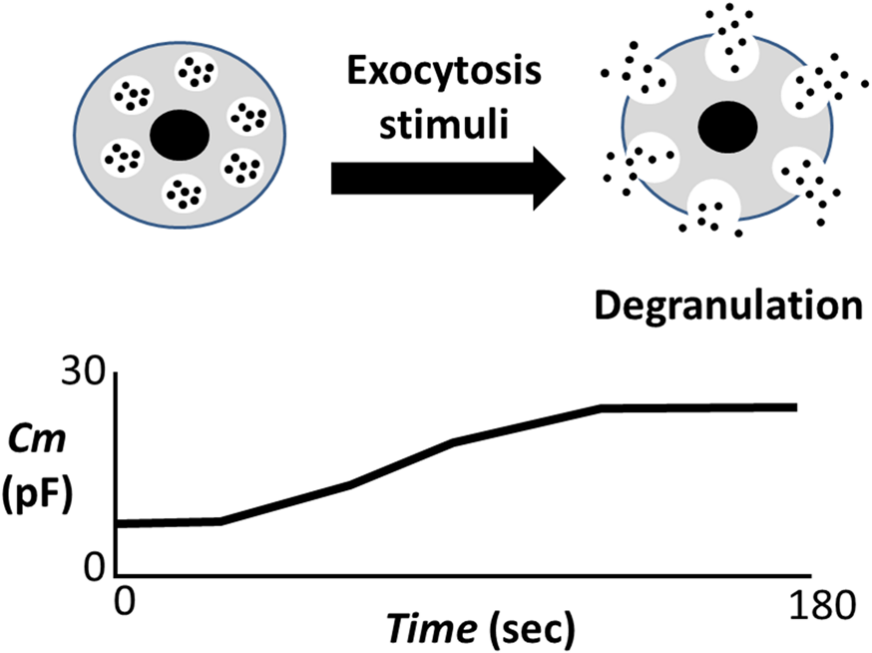
Degranulation from mast cells and the increase in membrane capacitance (Cm). Upon external stimuli for exocytosis, mast cells show more wrinkles on their cell surface and release secretory granules (degranulation), which is the process of exocytosis. As a result of exocytosis, membrane capacitance (Cm) gradually increases, representing the increase in the total cell surface area. Cm: membrane capacitance

**Fig. 2:**
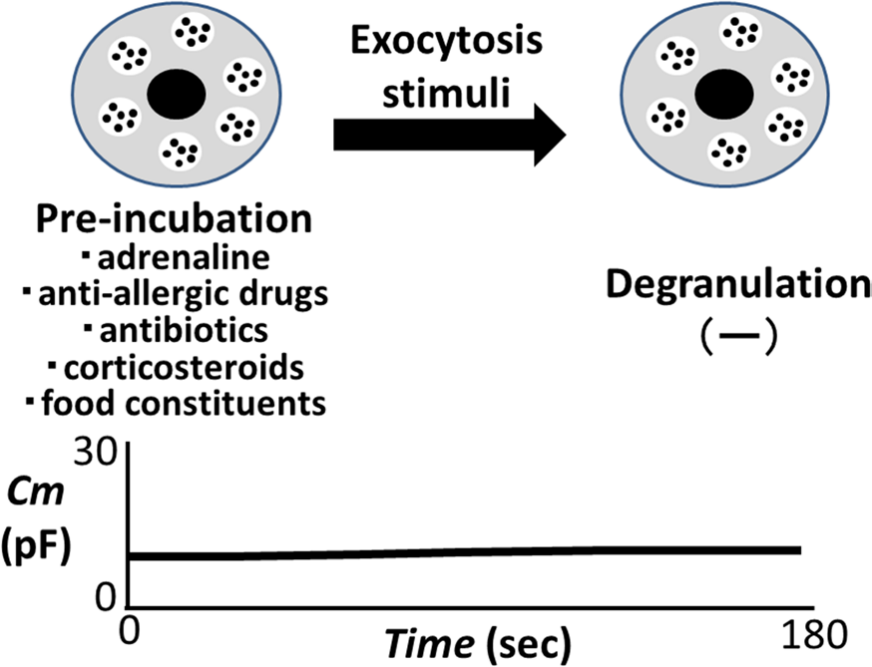
Prophylactic efficacies of adrenaline, commonly prescribed drugs or food constituents against anaphylaxis. Mast cells were pre-incubated with adrenaline, anti-allergic drugs (olopatadine, loratadine, tranilast and ketotifen), antibiotics (clarithromycin), corticosteroids (hydrocortisone and dexamethasone) or food constituents (caffeine and catechin) before exocytosis was induced. These drugs or the food constituents suppressed the increase in the membrane capacitance (Cm) and degranulation from mast cells, showing their prophylactic efficacy against anaphylaxis. Cm: membrane capacitance

Antihistamines or corticosteroids are also used as adjunctive therapy to anaphylaxis after adrenaline administration [20]. Regarding the prevention of anaphylaxis, some studies recommended the prophylactic use of these drugs [21, 22]. Nevertheless, the results have been controversial or the benefits were limited to the allergic reaction caused by anesthetic agents [23]. In previous studies, by quantifying the amount of histamine released from mast cells, inhibitory effects of drugs or substances on the activity of mast cells were indirectly determined [24, 25]. However, to accurately determine the mast cell-stabilizing properties of these agents, the process of exocytosis itself should be examined directly. In this regard, by continuously monitoring the changes in the whole-cell membrane capacitance (Cm) in mast cells, our recent patch-clamp study provided in vitro evidence that adrenaline dose-dependently inhibit the process of exocytosis [18]. This is based on the previous electrophysiological finding that the increase in the Cm represents the increase in the total cell surface area as a result of exocytosis [26] (Fig. 1). Therefore, the lack of increase in the Cm oppositely indicated the inhibition of exocytosis. In morphological analysis, being compatible with these findings, adrenaline actually suppressed the degranulation from mast cells dose-dependently, showing its effectiveness as a highly potent mast cell stabilizer (Fig. 2). In our experiments, mast cells that were freshly isolated from rat peritoneal cavity were initially pre-incubated with different concentrations of adrenaline for at least 10 min. Then, compound 48/80 (final concentration 10 µg/ml), the stimuli for exocytosis, were externally added. However, mast cells that were pre-incubated with relatively higher concentrations of adrenaline (100 mM, 1 mM) were almost totally prevented from being degranulated [18] (Fig. 2). These findings clearly indicated the prophylactic aspect of adrenaline in stabilizing mast cells. Additionally, in our study, high dose prazosin, a selective α_1_-adrenergic receptor antagonist, synergistically potentiated such prophylactic efficacy of adrenaline [18].

Using the same approach, our series of patch-clamp studies also revealed the inhibitory effects of anti-allergic drugs (olopatadine, loratadine, tranilast and ketotifen), antibiotics (clarithromycin) and corticosteroids (hydrocortisone and dexamethasone) on the exocytotic process of mast cells [26–29] (Fig. 2). In our studies, mast cell-stabilizing properties of these drugs were quantitatively determined by the suppressed value of the Cm, all of which showed dose-dependent inhibitory effects. In these studies, despite the external addition of exocytotic stimuli (compound 48/80), mast cells that were pre-incubated with these drugs were almost totally prevented from being degranulated, strongly showing their prophylactic efficacy in stabilizing mast cells [26–29]. Recently, we have additionally shown in in vitro study that both caffeine and catechin, that are main constituents of coffee and green tea, dose-dependently prevented the exocytotic process of mast cells [30]. In this study, we have further shown that low doses of catechin synergistically potentiated the mast cell-stabilizing property of caffeine.

COVID-19 vaccines containing PEG are contraindicated for individuals who have experienced prior allergic reaction to PEG [11]. Nevertheless, patients with mast cell diseases or individuals who have medical history of immediate allergic reactions to unidentified substances are allowed get vaccinated, as long as they are observed for 30 min following vaccination [11]. At present, regarding the prevention of COVID-19 vaccine-induced anaphylaxis, there are not enough clinical data to support the pretreatment effect of oral medications, such as antihistamines [11]. However, considering the findings obtained from our basic studies so far [26–30], the prophylactic use of the commonly prescribed medications (anti-allergic drugs, antibiotics and corticosteroids) or the food constituents (caffeine and catechin) may potentially be beneficial in preventing anaphylaxis caused by COVID-19 vaccination.

## Data Availability

The data used to support the findings of this study are available from the corresponding author upon request.
